# A pilot study investigating plasma pharmacokinetics and tolerance of oral capecitabine in carcinoma-bearing dogs

**DOI:** 10.1186/s12917-023-03805-y

**Published:** 2024-01-31

**Authors:** Sarah Wetzel, Janean Fidel, Dale Whittington, Nicolas F. Villarino

**Affiliations:** 1 Previously affiliated with the College of Veterinary Medicine, Washington State University, Pullman, WA USA; 2 Currently associated with SASH (Small Animal Specialist Hospital), North Ryde, NSW Australia; 3 College of Veterinary Medicine, Washington State University, Pullman, WA USA; 4 School of Pharmacy, University of Washington, Seattle, WA USA

**Keywords:** 5-fluorouracil, Adverse reactions, Canine, Capecitabine, Carcinoma, Chemotherapy, Pharmacokinetics

## Abstract

**Background:**

Capecitabine is an oral prodrug of the active metabolite 5-fluorouracil, which has been used effectively in human colorectal, head and neck, and mammary carcinomas. Capecitabine has several properties that make it an attractive treatment option for dogs: (i) it is relatively inexpensive, (ii) it has a short half-life in humans, allowing for rapid plasma concentration changes to be achieved with dosage adjustments, (iii) it is effective for treating carcinomas in humans, for which there are no widely-effective oral chemotherapy options in dogs, and (iv) it is thought to preferentially target cancer cells due to different expression of thymidine phosphorylase, thereby decreasing the risk of off-target side effects. However, capecitabine has not been widely explored as a chemotherapy agent for dogs. The goal of this study was to determine the plasma disposition of capecitabine in dogs following a single oral dose and to document any adverse events associated with capecitabine administration over the course of 5 weeks.

**Results:**

Capecitabine was well tolerated throughout the 5-week study period when administered to 5 dogs with naturally occurring carcinomas at 750 mg/m^2^ by mouth once daily for 14 consecutive days in a 3-week cycle. No dogs withdrew from the study due to adverse events or other causes. The median AUC_0-last_ was 890 h$\cdot$ng/ml (range 750-1100 h$\cdot$ng/ml); however, the maximum blood concentration and time to reach that concentration of capecitabine was highly variable after a single dose.

**Conclusions:**

Capecitabine appears well-tolerated as an oral chemotherapy agent for dogs with carcinomas, although individualized dosing may be necessary, and further studies are warranted.

## Introduction

Capecitabine is a fluoropyrimidine antimetabolite administered orally to humans for treatment of a variety of carcinomas including mammary carcinomas, head and neck tumors, and colorectal carcinomas [1–4]. After oral administration, capecitabine is absorbed through the intestines and undergoes initial metabolism in the liver [5]. It then undergoes further metabolism by thymidine phosphorylase at the tumor site to create the active metabolite 5-fluorouracil. Metabolites of 5-fluorouracil, synthesized intracellulary, are incorporated into cell DNA and RNA, resulting in apoptosis [5]. Because of differential expression of thymidine phosphorylase, the active metabolite of capecitabine is thought to localize more readily to tumor cells, reaching local tumor concentrations that are approximately 3-fold higher than in healthy tissue [6–8]. In humans, the peak plasma concentration for intact drug occurs after a median of 2 hours and then declines rapidly with a half-life of less than 1 hour [9, 10]. Approximately 75% of capecitabine metabolites can be recovered from human urine within 24 hours of oral drug administration [10]. This constitutes a rapid drug clearance or a relatively short half-life. From a clinical perspective, a short half-life is beneficial because it permits rapid plasma concentration adjustments when the dosage regimen is manipulated.

In humans, capecitabine is typically well-tolerated. However, dose limiting toxicities can include diarrhea, nausea, vomiting, fatigue, palmar-plantar erythrodysesthesia, ocular toxicity, or neurologic toxicity [11, 12]. Severe myelosuppression is not common [5]. When administered daily for 28 days, the maximum tolerated dose is 1,600 mg/m^2^ orally per day or, when administered daily for 14 days in a 3-week schedule, the maximum tolerated dose is 1,250 mg/m^2^ orally twice daily [13].

There is limited information about the use of capecitabine as an antineoplastic agent in dogs. In a recent study, response rate of carcinomas to a combination of carboplatin and the active metabolite of capecitabine (5-fluorouracil) in a gross disease setting was 43% with 3 complete responses [14]. Capecitabine has been explored, in combination with other immunosuppressive agents, to prevent allograft rejection in dogs. Most dogs tolerated this combination treatment, however, unpredictable neurologic and ophthalmologic adverse events were reported in a portion of dogs [15, 16].

Understanding the disposition of capecitabine in dogs would set the foundations for future pharmacokinetic and pharmacodynamics studies to establish safe and effective dosage regimens for treating dogs with carcinomas. One recent study evaluated the pharmacokinetic data of fluorocytosine, a related drug, but only in healthy dogs [17]. Our goal was to determine the plasma disposition of capecitabine, following a single oral dose of 750 mg/m^2^, administered to five client-owned carcinoma-bearing dogs admitted to the Washington State University Veterinary Teaching Hospital. We also aimed to document adverse events associated with administration of capecitabine for 14 consecutive days in a 3-week cycle over the course of 5 weeks when capecitabine was dosed at 750 mg/m^2^ orally once daily.

## Results

### Patient enrollment and initial monitoring

Five client-owned dogs from a variety of breeds with various carcinomas (see Table 1) were enrolled in the study. Comorbidities included skin allergies ($n=3$), hypothyroidism ($n=1$), hyperadrenocorticism ($n=1$), and osteoarthritis ($n=3$). Three dogs had previously received treatment for their tumors: 1 had radiation 14 months prior, 1 had IV mitoxantrone last given 1.5 months prior and oral chlorambucil last given 4 days prior, and 1 had Palladia last given 1 week prior. All dogs had been receiving nonsteroidal anti-inflammatory drugs (NSAIDs) prior to study enrollment and continued this treatment throughout the study without dose adjustments. Other medications that dogs received chronically while on the study included: CBD chews; levothyroxine; joint and vitamin supplements; Vetoryl and Denamarin; or Cosequin, maropitant, and mirtazapine. These medications did not prevent inclusion in the study as they were being administered prior to enrollment. All 5 dogs received their first dose of capecitabine without complication and were monitored for 24 hours in hospital. No dog had any adverse events during the first 24 hours after administration of the initial dose.

**Table 1:** Patient data

Patient	Breed/tumor type	Age (yr)	Gender	Weight (kg)	Capecitabine dosage	Week 5 response
Dog 1	Labrador retriever/	12	M	41.5	791 mg/m^2^	Progressive disease
	squamous cell carcinoma					
Dog 2	bulldog mix/	8	FS	33.9	762 mg/m^2^	Progressive disease
	thyroid carcinoma					
Dog 3	Labrador mix/	8	MC	41.0	798 mg/m^2^	Stable disease
	anal sac adenocarcinoma					
Dog 4	dachshund/	12	MC	12.8	818 mg/m^2^	Stable disease
	perianal carcinoma					
Dog 5	rough collie/	10	MC	43.2	772 mg/m^2^	Progressive disease
	urothelial carcinoma					

*yr* years, *kg* kilograms, *M* male intact, *FS *female spayed, *MC *male castrated

### Two and five-week capecitabine evaluation

All five dogs returned for re-evaluation at approximately 2 weeks and 5 weeks after starting capecitabine. Four of the five owners completed all requested journal information concerning adverse events while one returned only the first two days of the journal. No dog discontinued chemotherapy prior to the end of the study.

Adverse events were generally low grade and listed in Table 2. One dog had grade 1 constipation, while another had grade 1 lethargy. One dog had an approximately 1-minute syncopal episode with normal mentation before and afterwards. This event occurred in the first 2 weeks of treatment while under the care of a friend, who watched the pet for several days, so complete details could not be fully described. Further evaluation to determine the cause was declined by the owner. This dog continued capecitabine throughout the study period and no further episodes were reported. This dog also had grade 1 vomiting if their NSAID was given at the same time as the capecitabine but no vomiting was reported if the medication administrations were an hour apart. Intermittent grade 1 tenesmus and large bowel diarrhea were noted throughout the study in the dog with perianal carcinoma; these signs may have also been associated with his tumor and variable diet. One dog had grade 2 lethargy and inappetence for one day each; this dog also had grade 1 diarrhea, inappetence, and lethargy intermittently throughout the rest of the study. However, at final reimaging, they had bilateral hydroureters and hydronephrosis secondary to urothelial carcinoma progression, so these clinical signs may have been due to tumor progression rather than capecitabine side effects. No dogs had ocular changes during the study period. No dogs developed cutaneous lesions.

**Table 2:** Adverse events

Patient	Adverse event	Severity	Likelihood that capecitabine
			was cause of symptom
Dog 1	Azotemia	Grade 1	Possible
	Vomiting	Grade 1	Likely
	Syncope	Grade 3	Possible
Dog 2	Lethargy	Grade 1	Possible
Dog 3	Constipation	Grade 1	Possible
Dog 4	Tenesmus	Grade 1	Unlikely
	Diarrhea	Grade 1	Unlikely
Dog 5	Lethargy	Grade 2	Possible
	Inappetence	Grade 2	Possible
	Neutropenia	Grade 1	Possible
	Lethargy	Grade 1	Possible
	Inappetence	Grade 1	Possible
	Diarrhea	Grade 1	Possible
	Azotemia	Grade 2	Unlikely

A complete blood count (CBC) and serum chemistry test were performed on all dogs around enrollment, the 2-week, and the 5-week visits; see Tables 3 and 4. One dog with urothelial carcinoma had progressive grade 2 azotemia and grade 1 neutropenia at 5 weeks. All other hematologic changes were mild and deemed to be clinically insignificant.

**Table 3:** Hematologic parameters at pre-enrollment, 2 weeks after starting capecitabine, and 5 weeks after starting capecitabine. Bold numbers refer to values outside of the reference interval. Canine hematologic parameters at pre-enrollment, 2 weeks after starting capecitabine, and 5 weeks after starting capecitabine

		Dog 1			Dog 2			Dog 3			Dog 4			Dog 5		
Parameter	Normal range	Pre-enrollment	2 weeks	5 weeks	Pre-enrollment	2 weeks	5 weeks	Pre-enrollment	2 weeks	5 weeks	Pre-enrollment	2 weeks	5 weeks	Pre-enrollment	2 weeks	5 weeks
**WBC**	$4.5-16 \times 10^{3}/{\upmu }$L	8.6	13.1	7.6	6	5.6	4.9	10.9	10.6	9.8	5.6	5.7	5.4	6.9	5.2	**3.0**
**Bands**	$0-0.3 \times 10^{3}/{\upmu }$L	0	0	0	0	0	0	0	0	0	0	0	0	0	0	0
**Segs**	$2.8-13.4 \times 10^{3}/{\upmu }$L	6.364	10.611	5.472	4.620	4.032	3.626	7.085	6.572	6.664	4.424	4.161	4.374	5.106	3.432	**2.280**
**Lymphocytes**	$0.9-4 \times 10^{3}/{\upmu }$L	1.032	**0.524**	**0.836**	**0.840**	0.952	**0.784**	2.834	3.074	2.744	**0.504**	**0.798**	**0.594**	**0.414**	**0.728**	**0.210**
**Monocytes**	$0-1.3 \times 10^{3}/{\upmu }$L	0.688	0.917	1.14	0.480	0.224	0.392	0.872	0.424	0.294	0.448	0.684	0.324	0.621	0.728	0.150
**Eosinophils**	$0-1.2 \times 10^{3}/{\upmu }$L	0.516	1.048	0.152	0.060	0.392	0.098	0.109	0.53	0.098	0.224	0.057	0.108	0.69	0.312	0.360
**Basophils**	$0-0.1 \times 10^{3}/{\upmu }$L	0	0	0	0	0	0	0	0	0	0	0	0	0.069	0.069	0.069
**nRBC**	$0 /{\upmu }$L	**2**	0	0	0	0	0	0	0	0	0	0	0	0	0	0
**RBC**	$5.2-8.4 \times 10^{6}/{\upmu }$L	6.79	5.68	6.18	7.55	7.65	7.36	8.090	7.89	7.75	7.43	6.7	6.18	6.61	6.65	6.71
**Hemoglobin**	12-20 g/dL	15.4	13	13.8	16.9	17.2	16.1	19.6	18.7	18.4	16.9	15.5	14.1	15.6	15.4	15.5
**PCV**	36-56%	45	40	40	49	51	46	54	52	52	50	44	41	47	46	47
**MCV**	62-73 fL	66	69	65	65	67	63	66	66	67	68	66	66	71	69	70
**MCH**	22-26 pg	23	23	22	22	22	22	24	24	24	23	23	23	24	23	23
**MCHC**	35-38 g/dL	**34**	**33**	**34**	**35**	34	34	36	36	36	34	35	34	**33**	**33**	**33**
**RDW**	12-15%	15	15	15	13	14	14	12	13	13	12	12	13	12	12	12
**Platelets**	$160-500 \times 10^{3}/{\upmu } L$	443	403	481	212	202	233	283	278	299	487	**508**	473	293	286	250
**MPV**	7.4-13 fL	9.4	10.2	7.9	10.8	10.9	10.1	8.2	8.8	8.2	7.9	7.8	8.5	11.1	10.7	9.7

**Table 4:** Biochemical parameters at pre-enrollment, 2 weeks after starting capecitabine, and 5 weeks after starting capecitabine. Bold numbers refer to values outside of the reference interval. Canine biochemical parameters at pre-enrollment, 2 weeks after starting capecitabine, and 5 weeks after starting capecitabine

		Dog 1			Dog 2			Dog 3			Dog 4			Dog 5		
Parameter	Normal range	Pre-enrollment	2 weeks	5 weeks	Pre-enrollment	2 weeks	5 weeks	Pre-enrollment	2 weeks	5 weeks	Pre-enrollment	2 weeks	5 weeks	Pre-enrollment	2 weeks	5 weeks
**ALT**	0-100 U/L	31	62	31	60	54	46	87	78	91	**303**	**319**	**345**	84	55	77
**ALP**	0-96 U/L	46	52	50	**186**	**113**	**110**	**137**	**107**	95	**2064**	**2166**	**2272**	95	61	55
**Cholesterol**	134-359 mg/dL	234	217	204	257	275	287	272	312	321	244	280	312	326	284	308
**BUN**	9-26 mg/dL	**31**	**34**	**39**	19	20	21	17	17	18	**27**	14	17	**36**	**47**	**78**
**Creatinine**	0.8-1.6 mg/dL	1.6	1.6	**1.7**	0.8	0.9	1.1	0.9	1.1	1.2	1.0	**0.6**	0.9	1.4	1.4	**2.3**
**Glucose**	66-123 mg/dL	95	109	92	103	111	118	106	104	101	121	116	107	123	111	122
**Total Protein**	5.5-7.5 mg/dL	6.1	5.7	6.2	6.8	6.5	6.6	6.9	6.6	6.9	**7.9**	7.4	7.4	6.1	6.2	6.4
**Albumin**	2.9-3.8 mg/dL	**2.7**	**2.5**	**2.7**	3.1	3.2	3.3	**4.0**	3.9	**4**	3.6	3.5	3.6	3.4	3.5	3.6
**Globulin**	2.3-4.2 mg/dL	3.4	3.2	3.5	3.7	3.3	3.3	2.9	2.7	2.9	**4.3**	3.9	3.8	2.7	2.7	2.8
**Calcium**	9-11.3 mg/dL	10.7	10	10.9	10.3	10.1	9.8	10.2	10.3	10.9	**12.6**	**12.6**	**12.3**	10.7	10.2	**11.9**
**Phosphorus**	2.2-6.4 mg/dL	4.7	5.3	6.1	3.8	4	3.5	4.3	4.2	4	3.9	3.4	4	4.6	4.6	5.2
**T. Bili**	0 – 0.4 mg/dL	0.1	0.2	0.2	0.2	0.2	0.2	0.4	0.2	0.2	0.3	0.2	0.1	0.2	0.2	0.1
**Sodium**	149-158 mEq/L	150	**147**	**148**	149	**147**	**147**	**145**	**146**	**146**	**148**	**147**	149	151	151	149
**Potassium**	3.7-5.3 mEq/L	**5.5**	5.5	5.1	4.4	4.7	4.4	4.7	4.6	4.4	5.0	5.1	**5.4**	4.4	5	5.2
**Chloride**	112-119 mEq/L	113	**110**	**110**	112	112	**110**	**110**	**110**	**109**	**111**	**107**	**111**	119	115	112
**CO2**	15-26 mEq/L	23.2	25.1	22.8	25.5	21	25.4	20.7	21.3	20.8	**26.7**	25.9	25.9	16.3	22.6	22.8
**Anion Gap**	10-22 mEq/L	19.3	17.3	20.3	**15.9**	18.7	11.6	19	19.3	20.6	15.3	19.2	17.5	20.1	18.4	18.6
**HIL**		111	311	311	311	311	111	413	111	111	313	311	212	311	311	111

Tumor status was evaluated via physical examination and imaging on all dogs at the 5-week visit. Three dogs had progressive disease; see Table 1 for details. Two dogs, one with a perianal carcinoma and one with an anal sac adenocarcinoma, had stable disease based on computed tomography (CT) and ultrasound respectively. Both owners elected to continue capecitabine outside of the study and both dogs still had tumor control at the end of study data collection (150 days and 73 days post-first dose, respectively).

### Drug quantification and pharmacokinetic evaluation

**Fig. 1:**
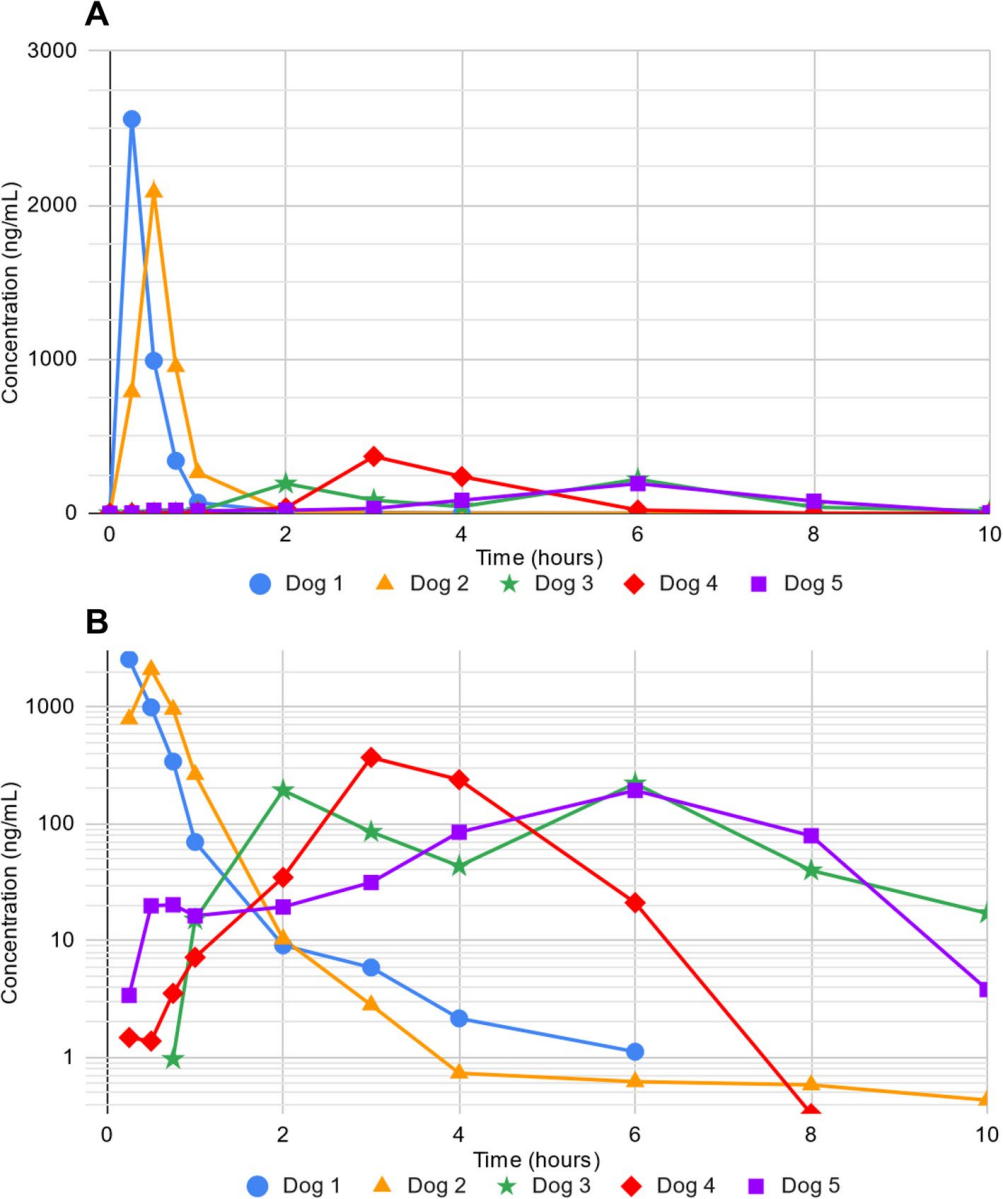
Capecitabine concentration in canine plasma as a function of time ($n=5$ dogs; oral dose target 750 mg/m^2^). **A** Linear plot of capecitabine concentration. **B** Semi-log plot of capecitabine concentration

All plasma samples were successfully analyzed for capecitabine concentrations. The median area under the concentration vs. time curve was 890 h$\cdot$ng/ml (range 750-1100 h$\cdot$ng/ml) up to last measurable concentration (AUC_0-last_) and 1000 h$\cdot$ng/ml (range 790-1100 h$\cdot$ng/ml) up to 6 hours (AUC_0-6 hrs_) for those with peaks in concentration before 6 hours. The maximum plasma concentration and time after oral dosing at which this maximum concentration was reached was highly variable between individuals (Fig. 1A). The median maximum concentration was 370 ng/ml (range 190-2600 ng/ml). The median time at which maximum plasma concentration was reached was 3 hours (range 0.25-6 hours; Table 5). No dog had capecitabine plasma concentrations above the level of detection (0.25 ng/ml) by 24 hours after dosing. Only 3 dogs had capecitabine plasma concentrations above the level of detection at 10 hours after dosing (Fig. 1B). The median plasma concentration of those dogs was 3.8 ng/ml (range 0.43-17 ng/ml).

**Table 5:** Pharmacokinetic parameters of capecitabine in dogs following single oral dose of 750 mg/m^2^. AUC values listed to 2 significant figures

Patient	Time to max concentration (h)	Observed max concentration (ng/ml)	Concentration at 10-h post capecitabine administration (ng/ml)	Area under concentration vs. time curve AUC_0-last_ (h⋅ng/ml)	Area under concentration vs. time curve AUC_0-6 hrs_ (h⋅ng/ml)
Dog 1	0.25	2600	BLLOQ	1000	1000
Dog 2	0.5	2100	0.4	1100	1100
Dog 3	6	220	17.2	890	–
Dog 4	3	370	BLLOQ	810	790
Dog 5	6	190	3.8	750	–
Median	3	370	3.8	890	1000

*BLLOQ* below lower limit of quantification (0.25 ng/ml), *h *hour

## Discussion

To the authors’ knowledge, this is the first study exploring capecitabine as an antineoplastic agent in a group of tumor-bearing dogs. In the current study, capecitabine was well tolerated: only 1 of the 5 carcinoma-bearing dogs had clinically relevant hematologic changes and these changes were deemed likely secondary to urothelial carcinoma progression. Clinical adverse events were typically mild and short-lived. One dog had a grade 1 nonfebrile neutropenia at 5 weeks. This clinically insignificant bloodwork change correlated to identification of bilateral hydroureters and hydronephrosis. Although this neutropenia could have been secondary to chemotherapy, sequestration as a result of urinary system inflammation or utilization as a result of secondary asymptomatic urinary tract infection were also possible etiologies. Only 1 dog had a possible neurologic adverse event, which involved a fainting-like episode that resolved spontaneously. The dog was reported by his owner, via second-hand account, to be normal immediately before and after the event and further workup was declined by the owner so the true cause of the event was unknown. This was in contrast with two studies in which capecitabine was utilized in an immunosuppressive regimen for renal allograft transplantation [14, 15]. In both of those studies, despite the lower dose of 50-250 mg/m^2^ twice daily, fatal neurotoxicity was observed in 25-29% of dogs. Other toxicities reported included nonfatal neurotoxicity in 57% of dogs in one study and superficial and pigmentary keratitis in 33% of dogs in the other study. The lack of ocular or neurologic toxicities seen in the current study suggests that the previously observed toxicity may have been due to the combination of immunosuppressive drugs, rather than secondary to the capecitabine alone. This correlates well with a study of dogs with inflammatory mammary carcinomas in which capecitabine was used in one dog at 750 mg/m^2^ once daily and no adverse reactions were observed [18].

The maximum concentration of plasma capecitabine in dogs and time at which this concentration was reached was highly variable between individuals in this study. The area under the concentration vs. time curve up to last measurable concentration (AUC_0-last_) ranged from 750 to 1100 h$\cdot$ng/ml. In humans the area under the concentration vs. time curve is reported to be 5.5-7.3 h$\cdot$mg/l (5500-7300 h$\cdot$ng/ml) [9], suggesting a higher dose may be needed in dogs to achieve therapeutic efficacy.

Possible causes of the observed pharmacokinetic variability in this study include differences in gastric emptying/intestinal absorption and differences in rate of conversion to the 5-fluorouracil. Previous studies on capecitabine have shown that food intake may result in variable capecitabine absorption [19]. Although all dogs were fasted for at least 8 hours prior to their first capecitabine dose and fed a commercial diet at the time of administration, it is unknown whether the length of the fast could result in absorption variation or whether types of food may alter absorption. All dogs ate at least 3 tablespoons of a commercial diet, however, the full amount eaten and the diet fed to dogs in this study differed depending on what the dog would eat in hospital and thus could account for some discrepancy. Additionally, dogs were allowed to maintain any medication/supplements that they were taking prior to study enrollment, which could result in synergistic or antagonistic effects. Although hepatic dysfunction, based on standard liver biochemistry results, has been reported in humans to alter capecitabine pharmacokinetics [10], only one dog in this study had increased ALT activity and that dog’s pharmacokinetic values did not differ from the other dogs’. Increased ALP activity was found in 3 dogs, but, again, this did not correlate with differing pharmacokinetic values. One dog was a known MDR-1 heterozygote and, interestingly, it had the lowest area under the concentration vs. time curve and maximum plasma concentration. Whether this dog could have also had other genetic mutations that would alter drug absorption/metabolism is unknown. The wide variability in pharmacokinetic parameters does suggest that individualized dosing may be needed for best efficacy and safety.

By 10 hours after the administration, only 3 dogs had quantifiable levels of plasma capecitabine and these concentrations were very low. No dogs had quantifiable levels by 24 hours, suggesting that all measurable capecitabine had been processed into its metabolites. This may suggest that increased dose intensity in the form of fewer/shorter breaks or decreased daily dosing interval may be beneficial for tumor control, particularly given the low incidence of significant side effects. Additional studies of dose intensity and dose intervals are warranted. In particular, twice daily dosing evaluation should be considered for future studies.

This study provides early support for continued evaluation of capecitabine as an antineoplastic agent in dogs, however, there are several shortcomings of this study. The sample size in this study was small, which –although standard for a pharmacokinetic study– may not reflect the larger population. Additionally, dogs were only followed for 5 weeks. It is possible that additional side effects or responses would have been identified had the study continued for a longer time. As described above, additional medications/supplements may have altered the pharmacokinetics of capecitabine. Since the standard population of carcinoma-bearing dogs are treated concurrently with NSAIDs and often have comorbidities requiring additional medications, the authors believe that the dogs in this study constitute an appropriate population sample. Finally, despite numerous attempts and various methods, we were unable to isolate 5-fluorouracil to confirm that capecitabine was metabolized into the active compound. Intermediate metabolites were not evaluated. A similar recent study was also unable to isolate 5-fluorouracil, which may suggest that this compound is particularly challenging to identify [17]. Ideally, additional larger-scale studies should be performed with quantification of the intermediate metabolites.

## Conclusion

Oral capecitabine, when dosed at 750 mg/m^2^ once daily, was well tolerated by carcinoma-bearing dogs and may serve as a reasonable starting dose for future conventional phase 1 dose finding trials. There is a possible trend towards high individual variability in pharmacokinetic parameters that deserves further evaluation. Further assessment of the efficacy of this drug is warranted.

## Methods

### Patient enrollment and initial monitoring

Five client-owned dogs that were seen by Washington State University Oncology Service between October 2020 and March 2021 for naturally occurring carcinomas in a gross-disease setting were enrolled in this study; see Table 1 for specific breed and tumor type. Enrollment criteria included cytology or histopathology diagnosis of a carcinoma, an expected survival of greater than 1 week, and informed owner written consent after declining alternative treatment options. No more than 2 weeks prior to enrollment, pets had to have a complete blood count (CBC), serum chemistry, and imaging of their tumor. Patients were excluded if their bloodwork did not reveal at least 2,500 neutrophils/$\mu$l. Liver or kidney enzyme elevation was not exclusionary to this study. Enrollment was restricted to dogs that could be dosed with capecitabine based on the available tablet sizes. Dogs were allowed to stay on any medications they were taking prior to enrollment but were not prescribed any additional new medications at the time of starting capecitabine. Although most dogs had been taking these medications for many months prior to enrollment, the duration of use was not standardized. This study was conducted in compliance with the ARRIVE guidelines and in accordance with Washington State University research guidelines and regulations.

All dogs were fasted for at least 8 hours prior to treatment and water was freely available throughout the study. A peripheral catheter was placed in all dogs for venous access in case of complications secondary to study drug administration. Dogs were fed a meal of commercial dog food and then received one oral dose of capecitabine (Accord, Durham, NC; NDC: 16729-0073-29 lot: PY03610 and NDC: 16729-0072-12 lot: M2006712), dosed at 750 mg/m^2^ and rounded to the nearest 150 mg or 500 mg tablet with no more than 10% variation above or below the intended dosage, within 15 minutes. This dose was based on half the tolerated human dose [13], which is a standard initial method for dosing chemotherapy in dogs if there is no published dosing information. Similarly, this dose had been previously utilized for a single dog with mammary carcinoma without adverse events reported [18]. Two ml of blood were collected either via direct venipuncture or from a peripheral catheter before drug administration and at 0.25, 0.5, 0.75, 1, 2, 3, 4, 6, 8, 10, and 24 hours post capecitabine administration. One dog had all samples collected from an IV catheter without discarding samples of blood to clear the catheter of previous saline flushes. Because this 0.4 ml of saline would dilute the total 3 ml blood volume drawn, this was accounted for by proportionally increasing the total concentrations of capecitabine obtained on analysis by the amount of catheter volume held. Blood samples were placed into EDTA tubes and then centrifuged at 1800$\times$g for 8 minutes to separate the plasma, which was then transferred into aliquots and stored at -80^∘^C until batch evaluation could be performed at University of Washington. Dogs were monitored via direct supervision and physical examinations throughout the day. They were offered a meal of commercial dog food at 8-10 hours after initial capecitabine administration. All dogs were hospitalized overnight under ICU care to monitor for neurologic, gastrointestinal, or other unexpected side effects based on reported human toxicities [5].

At 24 hours after the first capecitabine dose, dogs received an additional meal and a fluorescein eye stain with visual ocular examination for evidence of pigmentary keratitis or corneal changes was performed. Patients were then sent home with 14 doses of 750 mg/m^2^ capecitabine, to be given by mouth once daily starting that night or the next morning (based on owner preference). Owners were instructed to give capecitabine at a consistent time every day with a meal. They were also sent home with a daily journal to note energy, appetite, and stools on a 0 to 10 scale and report any other adverse events over the next 2 weeks.

### Two and five-week capecitabine evaluation

A recheck physical examination and CBC/chemistry was performed between 13 and 18 days after the first dose of capecitabine. Owners were questioned about any side effects or concerns at each recheck. All adverse events reported during recheck discussions or in the journal were graded using the Veterinary Comparative Oncology group adverse event grading system [20]. Dogs were then sent home with an additional 2 weeks of capecitabine to be restarted 1 week after the last dose of capecitabine. At approximately 5 weeks after starting capecitabine, a physical examination, CBC/chemistry, fluorescein eye stain, and recheck imaging were performed. As part of this exam, patient tumor status was evaluated using standard VCOG criteria [21]: complete response was defined as resolution of disease, partial response was defined as greater than $30\%$ decrease of tumor size, stable disease was defined as less than $30\%$ decrease of tumor size or less than $20\%$ increase in tumor size, and progressive disease was defined as greater than $20\%$ increase in tumor size or new lesions. If no tumor progression was noted, patients could continue capecitabine off the study.

### Drug quantification and pharmacokinetic evaluation

All canine plasma samples were shipped on dry ice to the University of Washington School of Pharmacy Mass Spectrometry Center laboratory for drug quantification. Drug quantification was performed on a Waters Xevo-XS with Waters Acquity I-Class UPLC chromatography unit using a Waters BEH C18 1.0$\times$100 mm, 1.7u column. Initial powdered stock of capecitabine (Sigma-Aldrich Inc., St. Louis, MO) and capecitabine-d11, the internal standard provided by the University of Washington School of Pharmacy’s Mass Spectrometry Center, were dissolved in dimethyl sulfoxide (Fisher, Hampton, NH) and further diluted to working stocks in methanol (Fisher, Hampton, NH). Working stocks of each were prepared and then mixed with blank canine plasma on a 1:1 ratio or a 1:10 ratio for sample quantification (higher dilution used if sample carryover was observed). For the initial method optimization, flow rate, acid quantity, and temperature were adjusted to allow for maximal peak separation, maximal amplitude, and minimal width in mobile phase. At final optimization, a run time of 8 minutes with a gradient mobile phase varying between 100% of 0.1% acetic acid (Fisher, Hampton, NH) in water and 100% of 0.1% acetic acid in methanol was utilized under the flow rate of 0.060 ml/minute at room temperature. Briefly, 20 $\mu$l plasma spiked with various concentrations of capecitabine was mixed with 20 $\mu$l capecitabine-d11 internal standard (10 ng/ml) and vortexed then 100 $\mu$l acetonitrile (Fisher, Hampton, NH) was added and the sample was vortexed and centrifuged at 18200 rcf for 5 minutes. Twenty-five $\mu$l supernatant was added to 25 $\mu$l H_2_O with 0.1% acetic acid then 10 $\mu$l was injected into the column. Once optimization was complete, controls and unknown plasma samples were run as a batch over 36 hours. For diluted samples, 2 $\mu$l of unknown plasma was mixed into 18 $\mu$l blank canine plasma with 20 $\mu$l capecitabine (10 ng/ml) and vortexed. This was added to 100 $\mu$l acetonitrile, vortexed, and centrifuged at 18200 rcf for 5 minutes. Then 25 $\mu$l supernatant was added to 25 $\mu$l H_2_O with 0.1% acetic acid and 10 $\mu$l was injected into the column. All standard curve concentrations (0.25-500 ng/ml) were analyzed followed by a blank prior to the unknown canine samples. Three quality controls (2.5, 25, and 250 ng/ml) and a blank were run between each set of canine samples.

The calibration curve was linear ($R^2=0.9997$) and the method was precise (coefficient of variation (CV) $\le 4\%$) and accurate (error $\le 11\%$). The dynamic range of the standard curve is 2.5 ng/ml to 375 ng/ml. The limit of quantification was 0.25 ng/ml and ranged up to 500 ng/ml.

Pharmacokinetic parameters were determined via noncompartmental analysis. Maximum plasma concentration and time at which maximum plasma concentration occurred were identified for each dog. The area under the concentration vs. time curve was calculated using the trapezoidal rule. That is, we estimated the AUC integral using the following sum:

1 $$\begin{aligned} \int _{0}^{T} f(t) \,dt \approx \sum \limits _{k=1}^{N} \frac{f(t_{k})+f(t_{k-1})}{2} \left( t_k - t_{k-1}\right) , \end{aligned}$$

where $t_0$, $t_1$, $\ldots$, $t_N$ is the discretization of the interval [0, *T*] (0 to time of the last measurable concentration or 6 hours).

## Data Availability

The data that support the findings of this study are available from the corresponding author upon reasonable request.
